# Tn*1* transposition in the course of natural transformation enables horizontal antibiotic resistance spread in *Acinetobacter baylyi*

**DOI:** 10.1099/mic.0.001003

**Published:** 2020-12-03

**Authors:** Julia Kloos, Pål J. Johnsen, Klaus Harms

**Affiliations:** ^1^​ Microbial Pharmacology and Population Biology Research Group, Department of Pharmacy, Faculty of Health Sciences, UiT The Arctic University of Norway, Tromsø, Norway

**Keywords:** *Acinetobacter baylyi*, natural transformation, Tn*1*, Tn*4401*, transposition

## Abstract

Transposons are genetic elements that change their intracellular genomic position by transposition and are spread horizontally between bacteria when located on plasmids. It was recently discovered that transposition from fully heterologous DNA also occurs in the course of natural transformation. Here, we characterize the molecular details and constraints of this process using the replicative transposon Tn*1* and the naturally competent bacterium *Acinetobacter baylyi *. We find that chromosomal insertion of Tn*1* by transposition occurs at low but detectable frequencies and preferably around the *A. baylyi* terminus of replication. We show that Tn*1* transposition is facilitated by transient expression of the transposase and resolvase encoded by the donor DNA. RecA protein is essential for the formation of a circular, double-stranded cytoplasmic intermediate from incoming donor DNA, and RecO is beneficial but not essential in this process. Absence of the recipient RecBCD nuclease stabilizes the double-stranded intermediate. Based on these results, we suggest a mechanistic model for transposition during natural transformation.

## Introduction

Horizontal gene transfer drives bacterial evolution through the acquisition of novel genetic material and accelerates the spread of adaptive traits such as antimicrobial resistance (AMR) between bacteria [1]. AMR in bacterial pathogens represents a growing public health concern [2] and there is an urgent need to increase our understanding of the basic mechanisms of AMR spread.

Conjugation, transduction and natural transformation are believed to be the main routes of intercellular gene transfer [3]. During conjugation, conjugative plasmids or conjugative transposons move into a recipient bacterium through cell-to-cell contact. Transduction includes the transfer of host DNA, mispackaged into bacteriophage particles during late infection, to a recipient cell. Natural transformation is the active uptake of free DNA from the environment and subsequent genomic integration [4]. Only bacteria that are competent for natural transformation can actively take up free DNA. Competence to undergo natural transformation was experimentally demonstrated in at least 80 bacterial species, both Gram-positive and Gram-negative [5]. The majority of the 12 global priority pathogens are naturally transformable, including those categorized as critically antibiotic-resistant: *Acinetobacter baumannii *, *Pseudomonas aeruginosa* and some *Enterobacteriaceae* [6, 7]. Natural transformation as a pathway for recruiting genetic variation, including AMR genes, was recently demonstrated between different species of the genus *Acinetobacter* [8] and different genera, for example from carbapenem-resistant *Klebsiella pneumoniae* to *A. baumannii* [9].

AMR determinants are frequently captured on mobile genetic platforms such as plasmids and transposons, and in associated mobilizable elements such as integrons, which all represent multidrug resistance-conferring units [10–12]. Transposons are widespread genetic elements in bacteria [13] and in other domains of life. They move intra- and intermolecularly between different genomic positions within a cell in a process called transposition [14]. Through transposition, transposons can be inserted into conjugative plasmids or into bacteriophages, which facilitates the mobility of non-conjugative transposons between cells [12]. Thus, transposon-embedded AMR determinants can be mobilized at multiple hierarchical genetic levels, and intermolecular movement of transposons between different plasmids or between plasmids and chromosomal locations also occurs within clinical pathogens [15–18]. Therefore, the investigation of transposon mobility across different bacterial hosts could aid the understanding of AMR spread.

Stable integration of horizontally acquired mobile genetic elements into the bacterial recipient genome is crucial for long-term inheritance and is facilitated by plasmid establishment or rearrangement events between donor and recipient DNA such as homology-based recombination, site-specific recombination, or transposition [12]. A recent study demonstrated the transposition of a Tn*21*-like transposon (Tn*3*-family) of *Salmonella enterica* serovar Typhimurium 490 into the chromosome of *Acinetobacter baylyi* ADP1 in the course of natural transformation [19]. Tn*21* insertion occurred independently at different loci in several transformants, and target site duplications (TSDs) at the insertion sites strongly suggested DNA recombination by transposition [19]. Since ADP1 harbours no transposons of the Tn*3*-family [20], these results indicated that the transposase was expressed from the incoming donor DNA. The authors concluded that a cytoplasmic, linear DNA double-strand formed after uptake, allowing gene expression and movement of the structural transposon [19].

In the present study, we used the Tn*3*-like replicative transposons Tn*1* [21] and Tn*4401* [22] as donor DNA to quantify and characterize transposition during natural transformation in molecular detail. As a recipient, we employed the naturally competent soil bacterium *A. baylyi* strain ADP1 also used by Domingues *et al.* [19]. ADP1 is transformable at high frequency by DNA from any source, including PCR products, which allows the detection and quantification of rare DNA recombination events [23, 24].

## Methods

### Bacterial strains and growth conditions

*A. baylyi* ADP1 strain BD413 Rp^r^ (wild-type) [25] and the derivative mutants Δ*recA::tetA* (JV37) [26], Δ*dprA::aacC1* (NH29) [27], Δ*recO* (KOM82) [27] and Δ*recBCD* Δ*sbcCD* (KOM45) [28] were employed as recipient strains in natural transformation assays and have been described previously. The Δ*xerC::nptII sacB* mutant was constructed as reported previously for *A. baylyi* deletion strains [29, 30]. Briefly, DNA sequences of about 1000 bp each upstream and downstream of *xerC* (ACIAD2657) in ADP1 (GenBank CR543861) were PCR-amplified using primers xerC-up-f/xerC-up-r (upstream segment) and xerC-down-f/xerC-down-r (downstream segment; all primer sequences are listed in Table S1 available in the online version of this article). The PCR products were inserted sequentially into the plasmid vector pGT41 upstream and downstream of a *nptII sacB* selectable marker gene pair. The resulting plasmid contained a Δ*xerC::nptII sacB* allele embedded into its natural flanking regions and was used to naturally transform ADP1. The resulting XerCD-deficient transformant (kanamycin-resistant) was confirmed phenotypically and by PCR (primers xerC-up-f/xerC-down-r and xerC-ctrl/xerC-down-r; Table S1). PCR assays were carried out with high-fidelity Phusion DNA polymerase (Thermo Scientific) and standard parameters except using an additional 10 % dimethylsulfoxide, or with DreamTaq DNA polymerase (Thermo Scientific).

*Escherichia coli* strains EC100 (Epicentre), DH5α [31] or SF8 *recA* [32] were employed as host strains for strain constructions and donor DNA preparations. Strains were grown in Luria–Bertani (LB) medium at either 30 °C (* A. baylyi *) or 37 °C (* E. coli *). For transformant selection, media were supplemented with antibiotics at the following concentrations: ampicillin, 100 mg l^−1^ (Tn*1* in pJK2, pJKH1, pJK7 and pTn*4401*); streptomycin, 40 mg l^−1^ (RSF1010); kanamycin, 10 mg l^−1^ (pKH80-PCR).

### Donor DNA preparations for natural transformation experiments

**Fig. 1.**
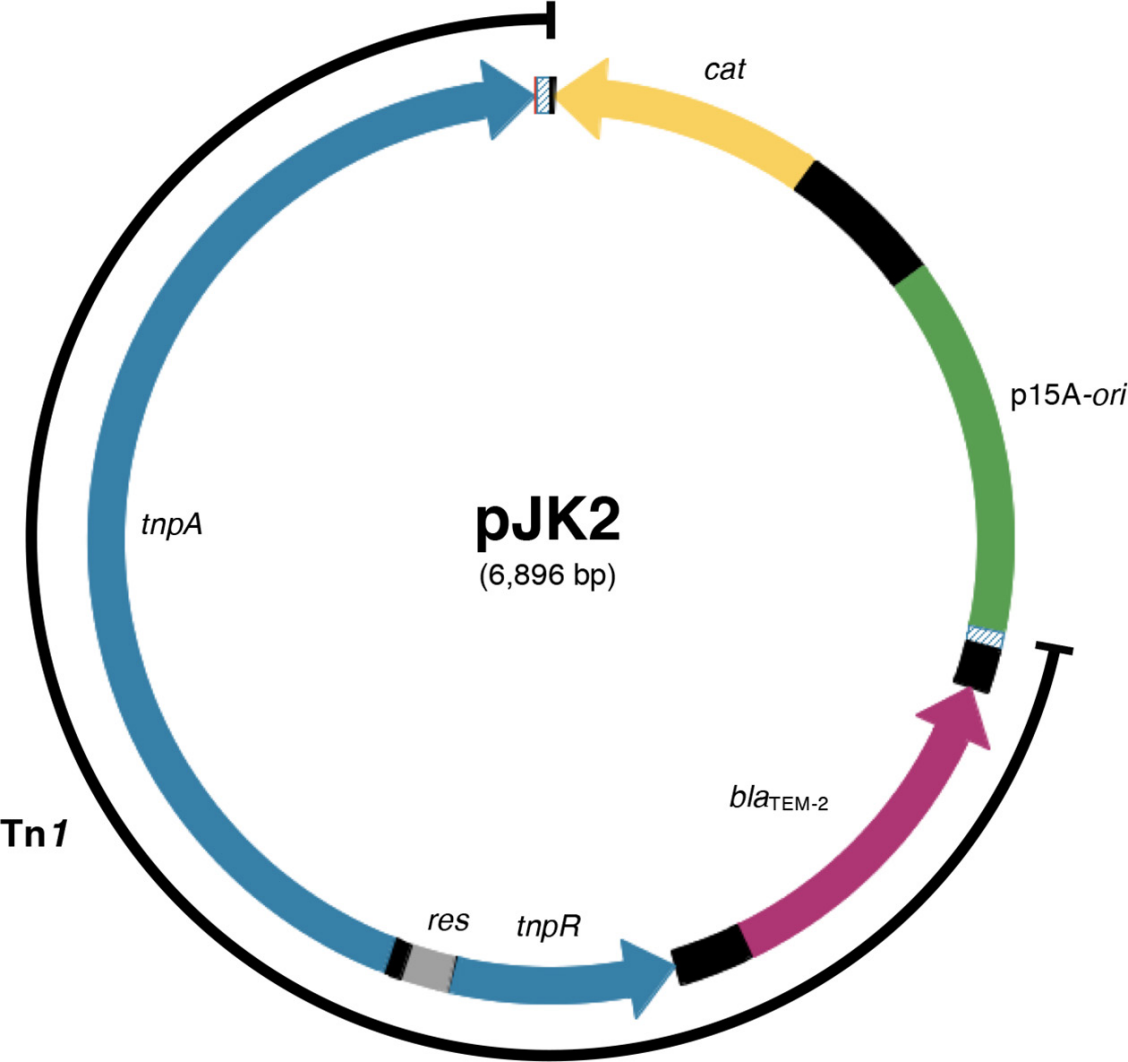
Map of plasmid pJK2. The plasmid backbone contains the p15A origin of replication (green) and the *cat* gene (yellow; chloramphenicol resistance) of the *E. coli* cloning vector pACYC184. Transposon Tn*1* (4.9 kb) was cloned from plasmid RP4. In Tn*1*, *tnpA* and *tnpR* (blue) encode the transposase and resolvase, respectively. Ampicillin resistance is conferred by *bla*_TEM-2_ (magenta). The transposon contains a *res* site for cointegrate resolution and is flanked by two terminal 38 bp inverted repeats (dashed). Neither Tn*1* nor pACYC184 display significant sequence identity with the *A. baylyi* genome.

Plasmid pJK2 (6896 bp; Fig. 1) was constructed as follows: transposon Tn*1* on plasmid RP4 (4951 bp; GenBank BN000925) was PCR-amplified from the left to the right border without TSDs using DNA from *E. coli* K12 J53 RP4 [German Collection of Microorganisms and Cell Cultures (DSMZ), DSM 3876] as template and primer Tn1-f+r (Table S1) as forward and reverse primer. The resulting PCR product was 5′-phosphorylated with polynucleotide kinase (Thermo Scientific) and ligated to a 1945 bp PCR product containing the p15A origin of replication and the chloramphenicol resistance gene *cat* of plasmid pACYC184 [33] (GenBank X06403; primers cat-r and p15A-r1; Table S1) using T4 DNA ligase (Fermentas). pJKH1 (carrying Tn*1*Δ*tnpA*; 4289 bp) was derived from pJK2 by digestion with *Eco*RV and circularization of the large fragment (T4 DNA ligase). The partial deletion of *tnpA* (869 internal of 1002 codons) was confirmed by restriction analysis. To construct plasmid pJK7 (Tn*1*Δ*tnpR*; 6399 bp), we PCR-amplified pJK2 excluding *tnpR* with primers pJK2-tnpR-del-f and pJK2-tnpR-del-r (Table S1). The PCR-product was 5′-phosphorylated (polynucleotide kinase) and circularized (T4 DNA ligase), and the deletion of *tnpR* was confirmed by Sanger sequencing. The plasmids RSF1010, pKH80 and pTn*4401* have been described previously [28, 34–36]. Plasmid donor DNA was isolated using the Qiagen Plasmid Extraction kit (Qiagen) and genomic donor DNA was purified with the GenElute Bacterial Genomic DNA kit (Sigma-Aldrich).

When PCR products were employed as donor DNA, PCR reactions were performed using high-fidelity Phusion DNA polymerase. Linear donor DNA substrate pJK2-PCR (6908 bp) was obtained by inverse PCR amplification of pJK2 with primers cat-f and p15A-ori-f-c (Table S1). This PCR product carried a 12 bp overlap at the ends. Donor DNA substrate pKH80-PCR (4760 bp) was PCR-amplified with primers sbcD-up-f and sbcC-down-r (Table S1) from plasmid pKH80. This substrate carried two approximately neighbouring 1.0 kbp DNA stretches from the *A. baylyi* chromosome covering parts of ACIAD0915 and ACIAD0918, interrupted in the centre by a 2.7 kbp *nptII sacB* marker gene cassette conferring kanamycin resistance. The PCR products were purified using the Qiaquick PCR purification kit (Qiagen). When indicated, pJK2-PCR was purified by agarose gel electrophoresis (Sea Plaque, LONZA) and subsequent recovery using the Qiaquick Gel Extraction kit (Qiagen).

### Natural transformation assays in liquid medium

Preparation of naturally competent cells of *A. baylyi* as well as natural transformation assays were conducted as described previously [29]. In brief, competent cell stocks were prepared by dilution of an overnight culture 1:100 into liquid LB and growth of that culture under aeration until logarithmic growth (1×10^9^ cells ml^−1^ determined with a haemocytometer) was reached. The cells were chilled, centrifuged for 10 min at 5000 ***g*** and 4 °C, and concentrated 1:10 in LB media containing 20 % glycerol (v/v). Finally, the cells were aliquoted and stored at −80 °C until further use.

For transformation assays, aliquoted competent cells were thawed on ice, diluted 1:40 in liquid LB to 2.5×10^8^ cells ml^−1^, and DNA was added at 100 ng ml^−1^ unless indicated otherwise. The assays were incubated under aeration for 90 min before the cells were chilled on ice, washed and resuspended in phosphate-buffered saline. Appropriate dilutions were plated on LB (recipient titre) and media containing selective antibiotics (transformant titre). LB plates were incubated for 24 h while transformant plates were incubated 48 to 72 h. Colonies were counted and transformation frequencies were calculated as transformants per recipient. If no transformant colonies were obtained, the limit of detection was calculated instead. In control experiments, recipient cells were incubated without donor DNA to identify resistant mutants arising and unselective and selective plating were performed according to the respective assay with donor DNA.

### Verification of transposition events

To identify transposition events, ampicillin-resistant transformant colonies were picked and restreaked three times on selective medium. In some cases, an intermittent cultivation step on non-selective medium was carried out. Typically, isolates were investigated after the final restreak by PCR for the presence of Tn*1* (primer Tn1-f+r) and pJK2 vector backbone DNA (primers cat-r/p15A-r1), or for the presence of Tn*4401* (primers KPC-A/KPC-B) and pTn*4401* vector backbone DNA (primers cat-r/p15A-r1), respectively (Table S1). Occasionally, ampicillin-resistant mutants arose during these cultivation steps, presumably through mutations. These isolates typically displayed an aberrant colony phenotype and were distinguished by the absence of transposon and pJK2 vector DNA in PCR analyses. These procedures clearly separated so-termed transposants from transient (unstable) transformants and mutants. Divergent transformant types were found in experiments using wild-type ADP1 with pJK7 donor DNA, or strain Δ*recBCD* Δ*sbcCD* with pJK2 donor DNA (see the Results and Discussion section for details).

To verify transposants, genomic DNA was isolated from stable ampicillin-resistant isolates that were PCR-positive for Tn*1* and PCR-negative for pJK2 vector backbone, using the GenElute Bacterial Genomic DNA kit (Sigma-Aldrich). Purified DNA was used as template for Sanger DNA sequencing (BigDye 3.1 technology; Applied Biosystems) as follows: 1 µg genomic DNA was mixed with 4 µl BigDye, 2 µl BigDye buffer and 10 µM of primer (either bla-ins-f or Tn1-tnpA-ins-f; Table S1) in a volume of 20 µl. Assays were denatured for 5 min at 95 °C, followed by 99 cycles of 30 s at 95 °C, 10 s at 55 °C and 4 min at 60 °C, and subsequently analysed on an Applied Biosystems 3130*xl* Genetic Analyzer (in-house sequencing facility). blast was used to identify both recombinant joints of Tn*1* with the chromosome of ADP1 (GenBank CR543861.1) and to verify the TSDs (Tables S2 and S3). The transposition frequency was calculated as transposants per transformant isolates multiplied by the transformation frequency.

### Characterization of target site sequences

**Fig. 2.**
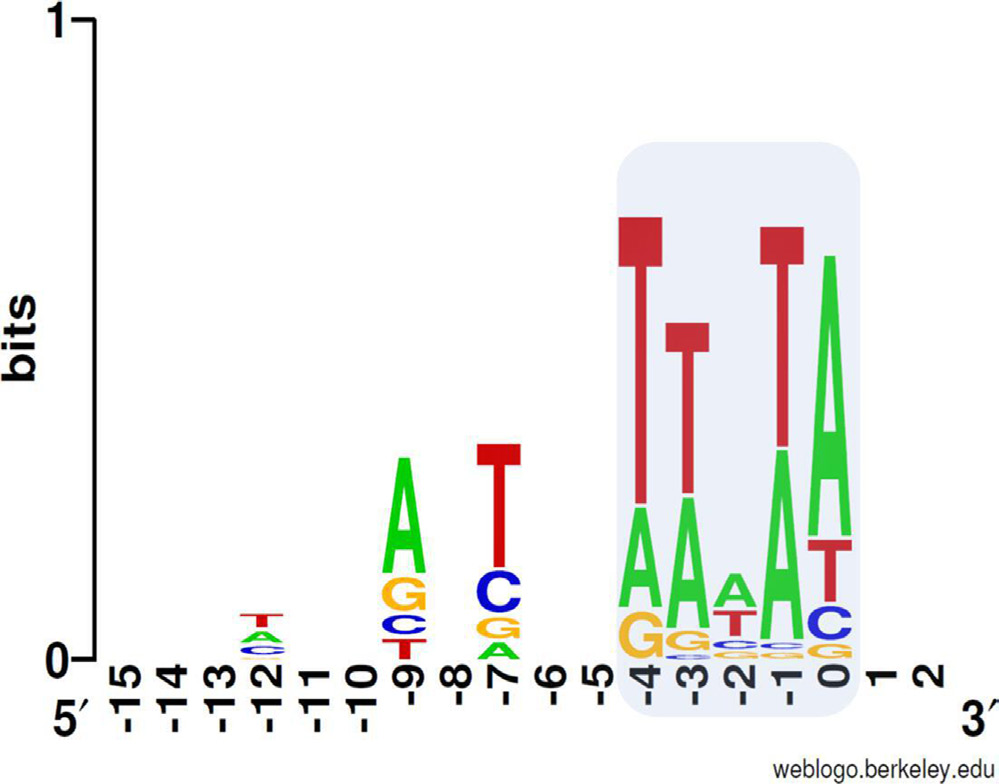
Consensus DNA sequence logo for Tn*1*-insertions into the chromosome of ADP1. Blue background colour indicates the target site duplication (TSD) consensus sequence at positions −4 to 0. Additional conserved positions around the TSD are shown. The TSD sequence motif (TTWTA; W: T or A) and preference for T at position −7 are typical for this transposon family. The logo was generated using WebLogo [37].

A consensus DNA sequence logo was generated by multiple sequence alignment and visualized using WebLogo software [37] (Figs 2 and Fig. S1) The aligned regions covered the 5 bp TSD plus 45 nucleotides upstream and downstream of the duplication (Table S4). The total number and positions of this TSD consensus sequence (TTWTA) were determined for the chromosome of ADP1 using Gene Construction kit v 4.0.3 (Textco BioSoftware, Inc.). Linear regression analysis of the TSD consensus sequence distribution over the chromosome of ADP1 was performed using GraphPad v 8.4.2 (GraphPad Software; *P* *** <0.001, ** <0.01, * <0.05).

### Susceptibility testing

The minimal inhibitory concentrations (MICs) were determined using gradient diffusion strips (Liofilchem) following the manufacturer’s instructions. Briefly, 0.5 McFarland solutions in 0.9 % saline (w/v) were prepared from freshly grown colonies on LB and spread evenly onto Müller–Hinton agar plates with a sterile cotton swab. A gradient strip for ampicillin was applied and plates were incubated at 30 °C for 18 h. Results were read at 100 % growth inhibition (*n*=1 per strain).

### Electroporation assays

Electrocompetent cells of *A. baylyi* were prepared as published for *E. coli* [38] with modifications. Briefly, a logarithmic culture of *A. baylyi* was grown at 30 °C in LB to 2.5×10^8^ cells ml^−1^. The cells were chilled, washed twice with ice-cold distilled water, and concentrated 500 times in 10 % ice-cold glycerol solution (v/v). A 40 µl aliquot was mixed with DNA (400 ng pJK2 or 200 ng RSF1010 DNA), transferred into a 2 mm gap electroporation cuvette and pulsed with 12.5 kV cm^−1^ (25 µF, 200 Ω) using a BioRad electroporator. Next, the cells were suspended in 1 ml prewarmed SOC (2 % tryptone, 0.5 % yeast, 10 mM NaCl, 2.5 mM KCl, 10 mM MgCl_2_, 10 mM MgSO_4_, 20 mM glucose) and aerated for 1 h at 30 °C. Appropriate dilutions were plated on LB (recipient titre) and LB containing antibiotics (transformants) and the plates were incubated at 30 °C for 16 to 20 h or 36 to 48 h, respectively.

## Results and Discussion

### Natural transformation by transposon-containing DNA

**Fig. 3.**
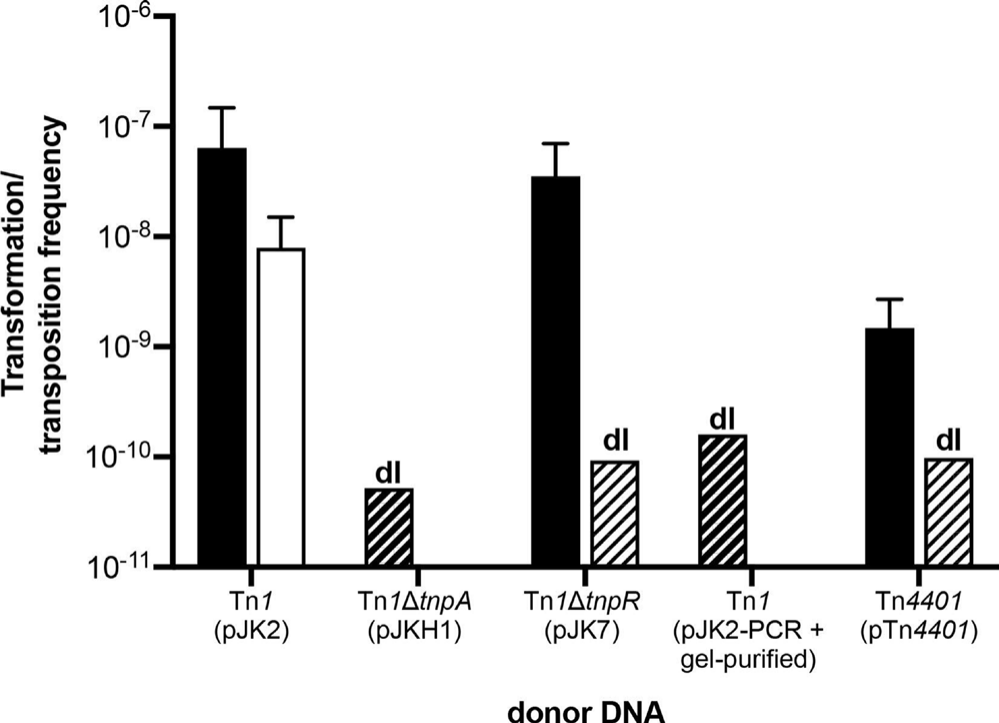
Frequencies of transformation (black bars) and transposition (white bars) in *A. baylyi* wild-type using different transposon-containing donor DNA substrates: pJK2 (Tn*1*; *n*=3), pJKH1 (Tn*1*Δ*tnpA*; *n*=4), pJK7 (Tn*1*Δ*tnpR*; *n*=3), pJK2-PCR (agarose gel-purified; *n*=3) and pTn*4401* (Tn*4401*; *n*=9). Bars represent the mean with standard deviation. Striped bars indicate the detection limit (dl) when no transformation or transposition events were observed.

We inserted the replicative transposon Tn*1* into the narrow-host-range plasmid vector pACYC184 that replicates in *Enterobacteriaceae* but not in *A. baylyi *. The resulting circular plasmid pJK2 (Fig. 1) was used as donor DNA to naturally transform *A. baylyi* ADP1 wild-type cells. Ampicillin-resistant transformant colonies were obtained at a frequency of (6.4±8.4)×10^−8^ per recipient cell (Fig. 3).

Analyses of resistant colonies revealed two distinguishable groups of transformants. In the first group, ampicillin resistance was stably maintained after repeated restreaking on selective medium and also after intermittent non-selective cultivation. PCR analyses showed the presence of Tn*1* but the absence of pJK2 vector backbone DNA. This result indicates that Tn*1* was chromosomally acquired by DNA uptake and transposition. In contrast, the ampicillin resistance was unstable in transformants of the second group, resulting in heterogeneous colony size and shape when restreaked. The resistance was generally lost after repeated purification on selective medium or after intermittent non-selective cultivation, and both Tn*1* and pJK2 backbone DNA could be PCR-amplified from cell material of the first, and with decreasing frequency of subsequent recultivations. This result suggests that transformants of group 2 received the pJK2 plasmid and became resistant through transient expression of *bla*_TEM-2_, but lost the plasmid and resistance with further cultivation due to the inability of pJK2 to propagate stably in *A. baylyi *.

To confirm that transformants of the first group occurred by transposition of Tn*1* into the recipient chromosome, we determined the DNA sequences upstream and downstream of Tn*1* by Sanger sequencing of genomic DNA. We analysed 20 group 1 isolates with circular pJK2 DNA and an additional 8 isolates obtained with linear pJK2-derivatives (Supplemental Information: Supplemental Results). Altogether, we identified 29 transposition events of Tn*1* into the chromosome of ADP1, including one isolate with two transposons (Table S2). In all transposition transformants (‘transposants’), Tn*1* was found as a chromosomal insert with a 5 bp TSD at the recombinant joints, which is a hallmark of transposase activity. The consensus sequence of the TSD is in agreement with previous reports on Tn*3*-family transposons (Fig. 2) [39, 40]. We did not identify fragments of pACYC184 vector DNA or indications of non-transposition recombination events. The MIC of ADP1 for ampicillin (2 μg ml^−1^) was increased to >256 μg ml^−1^ in eight randomly chosen transposants (Table S2). Together, these results confirm that the transposants have acquired Tn*1* during natural transformation through transposition.

The frequency of transposants using pJK2 donor DNA was (7.9±7.1)×10^−9^ (Fig. 3). To compare this transposition frequency to extra-chromosomal plasmid establishment or to homologous recombination during natural transformation, we used circular plasmid DNA (RSF1010) or linear homologous DNA (pKH80-PCR) in transformation assays with *A. baylyi* and obtained frequencies of (5.4±1.1)×10^−6^ and (1.3±0.5)×10^−4^, respectively. These data indicate that Tn*1* transposition in the course of natural transformation is about 680 times lower than plasmid aquisition and about 16 000 times lower than homologous recombination during natural transformation. Nonetheless, the transposant frequency was higher than illegitimate recombination frequencies with fully heterologous donor DNA in *A. baylyi* [24, 41]. We conclude that transposon spread through natural transformation can occur at biologically relevant frequencies, although reports showing this process *in situ* are currently lacking. The importance of transposant formation in the environment or for AMR spread in the hospital is unknown but probably low compared with conjugation.

### Chromosomal distribution of transposition events

**Fig. 4.**
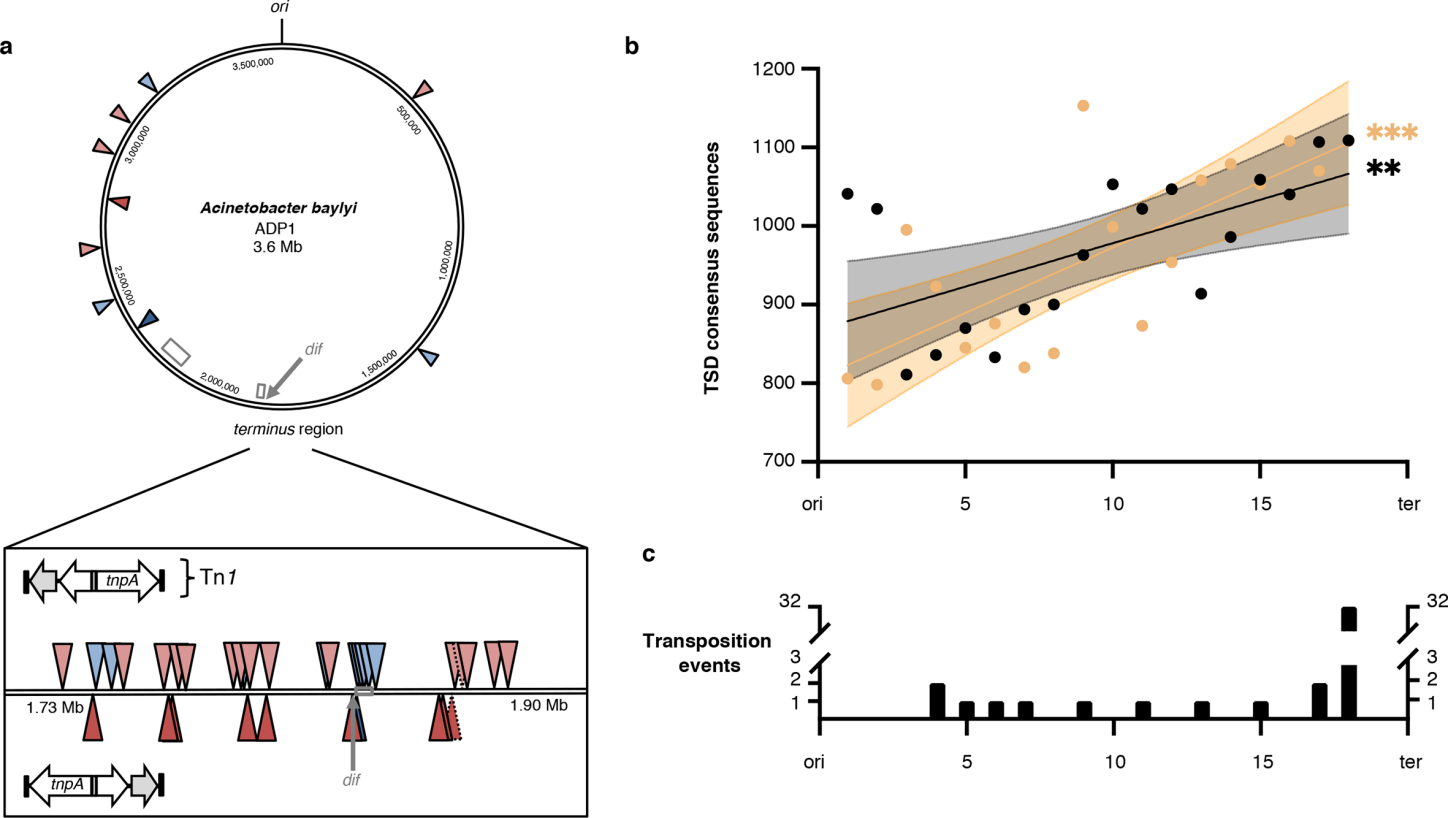
Characterization of Tn*1* insertion sites. (a) Chromosome map of ADP1 with a magnified section of ∼170 kbp (box) around the approximate terminus of replication (ter; G-C/G+C skew inversion site). This region contains 174 open reading frames, of which 12 are essential [42]. The two major prophage regions of ADP1 and the *dif* site for chromosome partitioning by XerCD are indicated by grey open boxes and grey arrows, respectively [20, 47]. Tn*1* insertions are marked as triangles (red, wild-type; blue, Δ*recBCD* Δ*sbcCD*). The orientation of Tn*1* is indicated by the colour intensity (light, transcriptional orientation of *tnpA* in sense; dark, *tnpA* in antisense). A dashed triangle edge specifies insertion sites in a double transposant. Thirty-four out of 43 insertions occurred around the terminus (24 in wild-type and 10 in the Δ*recBCD* Δ*sbcCD* strain). (b) Number and distribution of TSD consensus sequences across the chromosome of ADP1, which was bi-directionally grouped into 2×18 100 kb segments from origin (ori) to ter. The number of TSD consensus sequence hits per group is plotted along the ori–ter axis (black and orange, first and second replichore, respectively). For both directions, linear regression showed a significant increase in the number of hits (first replichore: *P*=0.0076, *R* ^2^=0.5544; second replichore: *P*=0.0004, *R* ^2^=0.3683; 95 % confidence intervals are indicated); lowest hit number: 798 (group 2, second replichore), highest hit number: 1153 (group 9, second replichore). (c) Number of experimentally observed transposition events per region.

All but one of the Tn*1* insertions occurred in nonessential genes [42] or in intergenic regions of the ADP1 chromosome (Tables S2 and S3). However, the distribution of insertions was nonrandom, and 24 of 29 transpositions clustered within a 170 kbp sector around the assumed terminus of replication (Fig. 4a) [20]. This distribution was supported by 14 additional unique Tn*1* insertions recovered from an *A. baylyi* Δ*recBCD* Δ*sbcCD* strain (Supplemental Information: Supplemental Results and Fig. 4a). To further characterize the distribution bias, we determined the total number of TSD consensus sequence sites and their positions across the *A. baylyi* chromosome. The TSD consensus sequence positions (*n*=34 863) followed a normal distribution, indicated by a small difference between the median and mean (0.16 %) relative to genome size. Although the incidence of TSD consensus sequence hits increases significantly along the origin–terminus axis of replication (Fig. 4b), even the highest increase in hit counts (30 %) does not explain why we observed more than 80 % of the verified Tn*1* insertions in a region representing only 5 % of the ADP1 chromosome (Fig. 4c).

A comparable transposon insertion bias has been observed previously, although not in the context of natural transformation. Tn*917* is a Tn*3*-like transposon in Gram-positive bacteria and preferentially inserts at the terminus in *B. subtilis* [43] and *Entercoccus faecalis* [44], but not in staphylococci [45, 46]. The bias is less strong in *B. subtilis* cells lacking functions involved in postreplicational chromosome segregation (RipX, SpoIIIE) or lacking the replication termination protein RTP [43]. In *B. subtilis*, *codV* and *ripX* encode the *dif*-directed chromosome partitioning recombinase often termed XerCD in Gram-negative bacteria [47]. We hypothesized that the absence of XerCD would alter the observed insertion bias of Tn*1* in *A. baylyi *. We naturally transformed an ADP1 Δ*xerC* mutant by pJK2 donor DNA but did not obtain any transformants (detection limit: 2.2×10^−10^; *n*=3). The reason for this result is unclear. Further experimental investigation was impeded by the genetic limitations of the employed recipient strain: *A. baylyi* carries no gene homologous to *rtp* (*tus* in *E. coli *) [24], and the orthologue of *spoIIIE* (*ftsK*) is essential in *A. baylyi* [42]. Using RSF1010 as donor DNA for the *A. baylyi *Δ*xerC* strain, the transformation frequency was (1.4±0.7)×10^−6^ (*n*=3), which was only four times decreased compared to the wild-type.

Finally, we hypothesized that the distribution of Tn*1* insertions would be different using artificial transformation (electroporation). During electroporation, circular double-stranded DNA is directly delivered to the cytoplasm. However, no ampicillin-resistant isolates were obtained after electroporation of *A. baylyi* by circular pJK2 DNA (detection limit: 3.6×10^−10^; *n*=3). In contrast, electroporation by RSF1010 (*n*=1) resulted in streptomycin-resistant transformants at a frequency of 5.6×10^−5^, which was 10 times higher than the frequency observed with natural transformation of *A. baylyi *. The reason for the distribution bias of transposition events remains unknown.

### Requirement of transposon genes for transposition

Tn*1* carries two genes involved in transposition: *tnpA* is the transposase gene, and *tnpR* encodes the resolvase for cointegrate intermediates created by TnpA. TnpR also acts as a repressor for both *tnpA* and *tnpR* [48] (Fig. 1). We hypothesized that transposition and cointegrate resolution during natural transformation were conferred by the *tnpA* and *tnpR* gene products encoded by the donor DNA and that deletion of these genes would decrease transposant frequency. We removed the *tnpA* gene from Tn*1* of pJK2, and natural transformation of *A. baylyi* by the resulting plasmid pJKH1 yielded no ampicillin-resistant transformants (detection limit: 5.2×10^−11^; Fig. 3), confirming that *tnpA* of Tn*1* but no recipient functions act as transposase for Tn*1*.

We also used plasmid pJK7 (carrying Tn*1*Δ*tnpR*) as donor DNA for the transformation of *A. baylyi* and the transformation frequency was similar to that obtained with pJK2 [(3.5±3.5)×10^−8^; Fig. 3]. We PCR-screened 150 transformant isolates that were stably resistant after three consecutive streakouts and found that Tn*1* and pJK2 vector backbone was detectable in all isolates. This transformant type was not detected with pJK2 but is consistent with unresolved cointegrate intermediates. We conclude that in the absence of TnpR, the derepressed TnpA efficiently recombined Tn*1* with the recipient and formed cointegrates that remained unresolved. Recipient functions such as RecA may contribute to their eventual resolution, but, taken together, these findings indicate that the main resolvase is the TnpR encoded by the donor DNA.

### Formation of double-stranded cytoplasmic intermediates

Taken together, the presented results demonstrate that *tnpA* and *tnpR* genes as well as *bla*_TEM-2-_ in the transient transformants of group 2 described above are expressed after uptake of DNA into the cytoplasm. Moreover, our data suggest that a circular intermediate is involved in the first (cointegration) step of transposition. However, the donor DNA is transported as linear single-strands into the cytoplasm during natural transformation [49, 50]. We hypothesized that the uptake of two complementary DNA single-strands is required for double-strand formation by hybridization. The resulting linear DNA double-strands, however, would be susceptible to degradation by exonucleases such as RecBCD [51, 52-]. Subsequent circularization by annealing at overlapping ends, followed by fill-in DNA synthesis or gap repair, would protect these double-strands from exonucleolytic degradation [53].

**Fig. 5.**
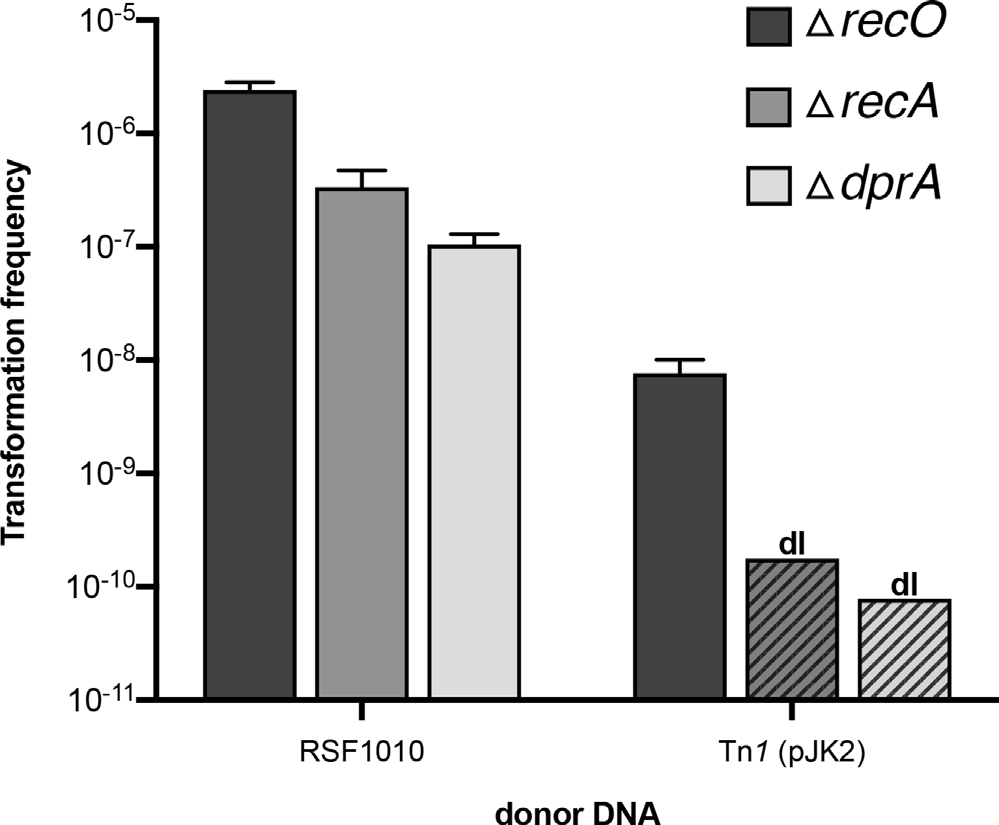
Transformation frequencies of *A. baylyi* mutant strains (Δ*recO*, Δ*recA* or Δ*dprA*) by circular donor DNA substrates (RSF1010 or pJK2). Bars represent the mean with standard deviation from three to five experiments. Striped bars indicate the detection limit (dl) when no transformation or transposition events were observed.

In *B. subtilis*, *recO* is required for efficient plasmid transformation [54], which can be explained by the activity of RecO to hybridize complementary DNA single-strands [55]. To investigate the role of RecO in double-strand formation in *A. baylyi,* we transformed a Δ*recO* strain by circular pJK2 DNA. The resulting frequency of ampicillin-resistant transformants was eight times lower than in wild-type *A. baylyi* [(7.7±2.4)×10^−9^; Fig. 5]. With RSF1010 as donor DNA, the frequency was (2.4±0.4)×10^−6^, which was two times lower than in wild-type *A. baylyi* (Fig. 5).

Unlike *recO*, the *recA* gene is not required for plasmid transformation of *B. subtilis* [54], suggesting that RecO is sufficient to restore circular plasmids from two complementary single-strands in this organism. We tested whether this was also the case in *A. baylyi *. In a Δ*recA* mutant transformed by circular RSF1010 DNA, plasmid transformants occurred at a frequency of (3.4±1.3)×10^−7^ (Fig. 5), which was ~16 times lower than in wild-type *A. baylyi *. With pJK2 as donor DNA, no transformants were obtained (detection limit: 1.8×10^−10^; Fig. 5). This result suggests an unexpected role of RecA in plasmid circularization in *A. baylyi *.

The DprA (DNA processing A) protein is thought to load incoming DNA single-strands with RecA protein [56]. In many naturally competent bacteria, deletion of *dprA* abolishes or severely reduces natural transformation [27, 57–60]. We investigated whether natural transformation by circular extrachromosomal DNA was affected in an *A. baylyi* Δ*dprA* mutant. Using pJK2 as donor DNA, the transformation frequencies of the Δ*dprA* strain dropped below detection limit (7.8×10^−11^) (Fig. 5). With RSF1010, the transformation frequency was (1.4±0.2)×10^−7^ and thus approximately 50 times lower than that of the wild-type (Fig. 5). These frequencies are comparable with those using the Δ*recA* strain as recipient. The results support the assumption that DprA acts upstream of the RecA recombination pathway. They also demonstrate that plasmid transformation (RSF1010) is not abolished in the absence of DprA.

Our findings show that deficiencies of RecA, DprA and, to a lesser degree, RecO reduce transformation of circular extrachromosomal DNA in *A. baylyi *, suggesting a role of these functions in plasmid circularization. In our experiments, the non-replicative plasmid pJK2 generally yielded poorer transformation frequencies than the replicative plasmid RSF1010. We cannot exclude the possibility that some or all of the investigated genes also modulate the transposition efficiency in addition to double-strand conversion and circularization.

Next we tested whether circularization is a requirement for transposons to jump during natural transformation. We used an inverse PCR product of pJK2 as donor DNA with Tn*1* approximately in the centre (pJK2-PCR). The substrate was agarose gel-purified to eliminate all traces of contaminating template DNA (confirmed by PCR; Supplemental Information: Supplemental Results). Using this linear DNA substrate for transformation of wild-type *A. baylyi *, no ampicillin-resistant transformants were obtained (detection limit: 1.6×10^−10^, Fig. 3), suggesting that pJK2-PCR is insufficient to produce both transient transformants and transposants, presumably due to the cytoplasmic instability of linear double-stranded intermediates.

### Antagonistic activity of RecBCD

**Fig. 6.**
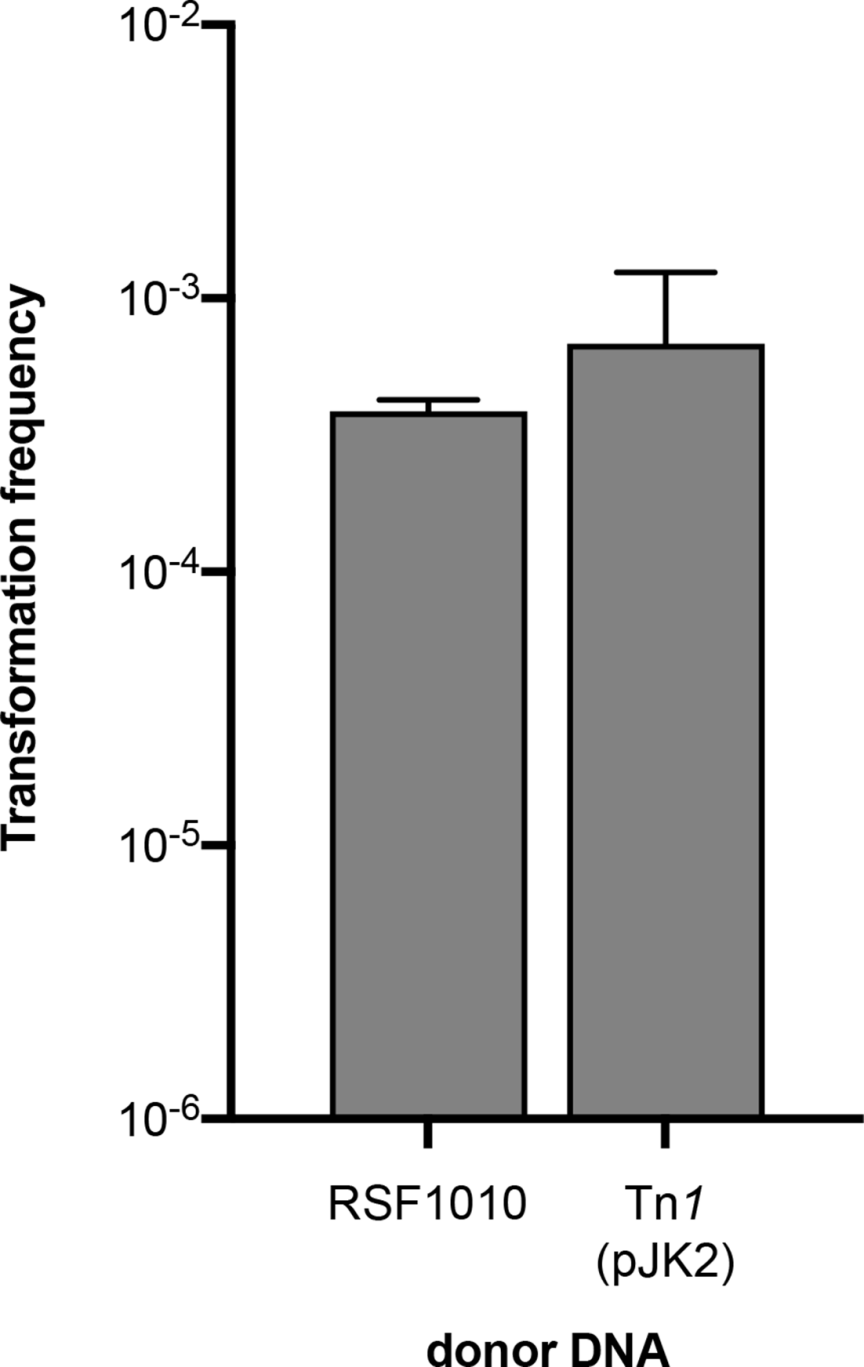
Transformation frequencies of the Δ*recBCD* Δ*sbcCD* strain using plasmid DNA substrates (RSF1010 or pJK2; *n*=5). Bars represent the mean with standard deviation.

Cytoplasmic linear double-stranded DNA is target for degradation by the RecBCD exonuclease in many bacteria [52] and the absence of the nuclease may protect linear intermediates and enhance plasmid circularization. In *A. baylyi *, RecBCD is thought to destroy donor DNA following uptake into the cytoplasm after the creation of double-stranded ends during homologous recombination [30]. However, in the context of plasmid transformation, in *B. subtilis* strains lacking AddAB, the functional equivalent of RecBCD, transformation frequencies are somewhat reduced [61]. To investigate the effect of RecBCD deficiency on plasmid transformation in *A. baylyi *, we transformed a Δ*recBCD* Δ*sbcCD* strain by RSF1010 and obtained a 70 times increased transformation frequency [(3.9±0.4)×10^−5^] compared with the wild-type (Fig. 6). We also transformed the Δ*recBCD* Δ*sbcCD* strain by Tn*1*-containing donor substrates. Using a gel-purified linear pJK2-PCR substrate, no transformants were obtained (detection limit: 4.9×10^−10^; *n*=3), strongly suggesting that a circular intermediate is required for transposition and transformation. With circular pJK2 donor DNA the frequency of ampicillin-resistant transformants was (6.8×±5.6)×10^−4^ (Fig. 6), which was four orders of magnitude higher than that of the wild-type. Analysis of 40 ampicillin-resistant isolates revealed stable resistance even after repeated recultivation. PCR analyses verified the presence of Tn*1* and pJK2 vector backbone DNA in all isolates and circular pJK2 plasmid DNA could be isolated from cell material of restreaks, indicating extrachromosomal DNA rather than cointegrates. No transposants were identified during our standard screening, but a transformational screening approach revealed 14 unique chromosomal insertions (Supplemental Information: Supplemental Results and Table S3). These results were unexpected, and we concluded that established pJK2 replicated relatively stably in *A. baylyi* in the absence of RecBCD. The parental plasmid of pJK2, pACYC184 [33], propagates as a theta replicon in *E. coli* and in other *Enterobacteriaceae* [62]. It is possible that absence of RecBCD allows rolling circle replication of pACYC184 in *A. baylyi *, as in *E. coli* RecBCD*-*deficient mutants [62]. In wild-type *E. coli *, the exonucleolytic activity of RecBCD is thought to degrade the nascent multimers of the rolling circle, forcing the plasmid into theta replication. In contrast, loss of RecBCD allows rolling circle replication [62].

### A model for natural transformation by transposons

In this study we experimentally verify that bacteria can acquire transposons horizontally through transposition in the course of natural transformation, as observed previously for a Tn*21* transposon [19]. In control experiments, we investigated the circularization of a replicative plasmid in *A. baylyi* during transformation, which has been studied before in *B. subtilis* [54]. Our results indicate differences in plasmid transformation between the two species. In contrast to our results, absence of RecA in *B. subtilis* did not affect plasmid establishment; instead RecO protein was required for that process [54].

**Fig. 7.**
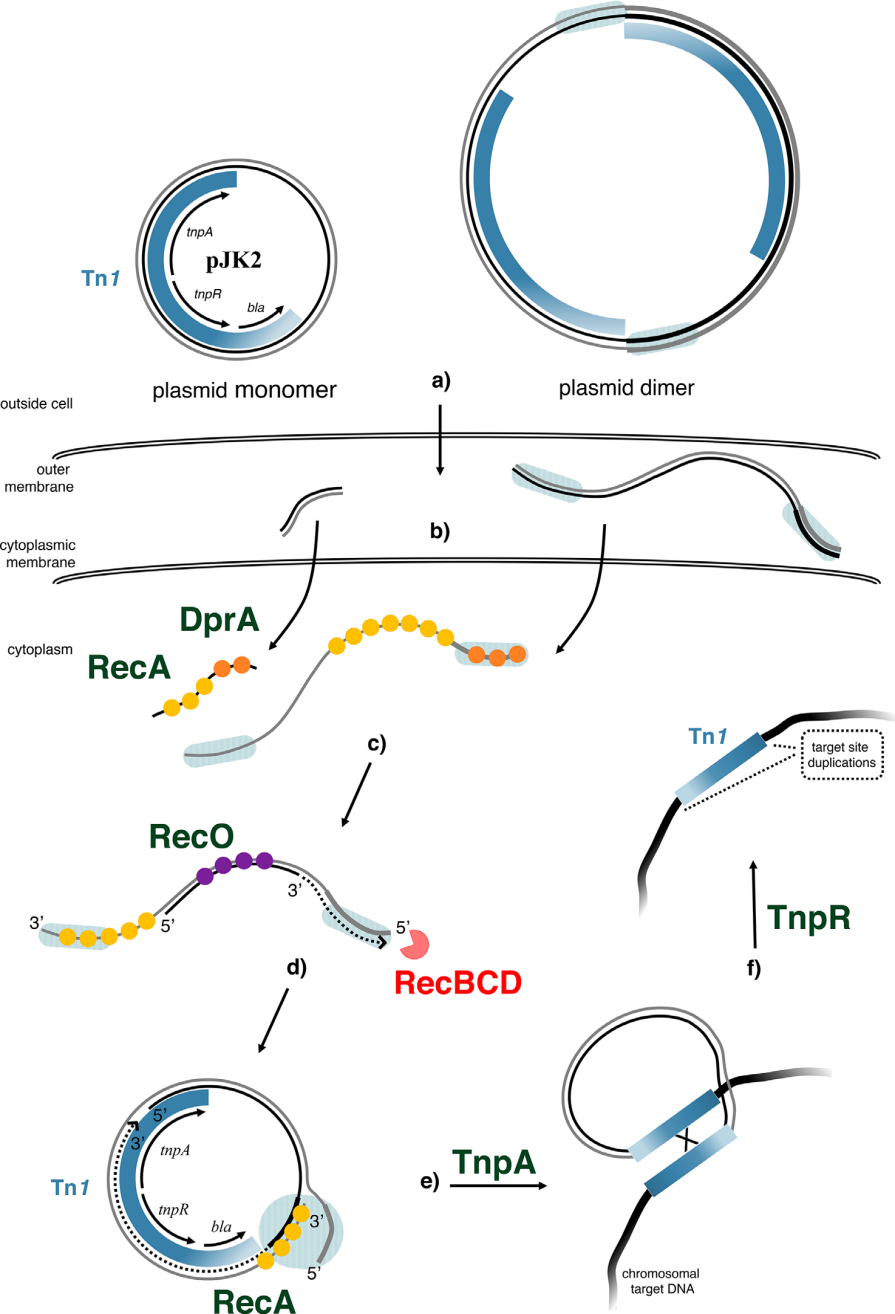
Model of Tn*1* transposition during natural transformation of *A. baylyi *. Proteins with beneficial or necessary functions are represented in green, and proteins with antagonistic functions are red; exemplary sequence segments providing homology are indicated by a shaded background. (a) Circular monomeric as well as sporadic dimeric forms of plasmid DNA are available for uptake into the periplasm. (b) DNA single-strands are taken up into the cytoplasm and protected from degradation by DprA (orange). DprA also loads RecA (yellow) onto single-stranded DNA. (c) RecO (purple) is involved in the annealing of DNA single-strands [55], in initiating or improving gap repair as part of RecFOR or RecOR [63, 64], or both. The linear double-stranded molecule is susceptible to degradation by RecBCD exonuclease (red). (d) The RecA-loaded DNA 3’-single-strand end invades the double-stranded fragment at its terminal homology, and circularization of the donor plasmid is completed by DNA repair and gap synthesis. (e) From the established circularized plasmid, the genes necessary for transposition (*tnpA*, *tnpR*) are expressed, and TnpA together with a recipient DNA polymerase form a transposon–target DNA cointegrate and generate the TSD. (f) TnpR resolves the cointegrate, and the recipient chromosome contains now a copy of Tn*1*.

Based on our findings, we propose a model for plasmid transformation and transposition in *A. baylyi* (Fig. 7). We suggest that sporadic dimeric forms of plasmid DNA with redundant ends are the main substrate for cytoplasmic donor DNA circularization. To initiate circularization, a small single-strand fragment is annealed with a large dimeric complementary strand with 5′- and 3′-overhangs. DNA fill-in synthesis converts the 5′-overhang into a double-strand (using the 3′-recessed end as primer), while the 3′-overhang is charged with RecA protein. It is consistent with our findings that DprA has a RecA-loading function [56]. The resulting nucleoprotein filament can undergo homology search. When it finds the homology of the redundant double-stranded end, the result is a circular intermediate with a displaced strand, and textbook DNA repair and gap synthesis generate a circular double-strand. This model is supported by lack of transformants with linear pJK2-PCR as donor DNA, since this substrate did not contain dimeric DNA molecules. The resulting circular double-stranded intermediate is temporally protected from DNA degradation (Fig. 7). Finally, the *tnpA* and *tnpR* genes are expressed and can lead to transposition of Tn*1*.

Is circular double-stranded DNA in general a necessary requirement for transposition in the course of natural transformation? Probably not. Domingues *et al.* obtained transposants using chromosomal DNA for natural transformation of *A. baylyi* [19]. Hypothetically, such DNA can form circular intermediates at direct repeats, and transposons surrounded by direct repeats may be stabilized through DNA circularization. It is also conceivable that Chi sequences surrounding the transposon protect the double-stranded intermediate from exonucleolytic degradation [51].

To put the results of this study into broader perspective, we employed a published transposon-containing plasmid as donor DNA for transformation of ADP1. The pACYC184 derivative pTn*4401* (15.6 kbp) [35] carries the replicative transposon Tn*4401* (~10 kbp) of the Tn*3-*family that contains the carbapenemase gene *bla*_KPC-2_ [22]. Ampicillin-resistant transformants formed at a frequency of (1.5±1.2)×10^−9^ (Fig. 3), which was 40 times lower than the frequency observed with pJK2 DNA. Among 60 investigated transformants, no transposants were found (detection limit: 9.8×10^−11^). Several explanations for the lack of transposition of Tn*4401* are conceivable: the MIC conferred by KPC-2 may be too low to detect transposants in this organism. The plasmid size possibly reduces the dimer/monomer ratio. Alternatively, the repression of *tnpA* may be tighter in Tn*4401* than in Tn*1*. Taken together, this result shows that additional constraints exist for transposition during natural transformation, and further investigations are needed.

In conclusion, we showed that transposition of Tn*1* in the course of natural transformation occurs at biologically relevant frequencies. Our results open up the possibility that transposable elements can even spread from dead cells, where fully heterologous, transposon-containing free DNA can transform naturally competent bacteria, albeit at low frequencies. Transposons play an important role in the dissemination of multi-drug resistance, and the identification of both the transferred resistance genes and the genetic context that was transferred is crucial to understand AMR spread.

## Supplementary Data

Supplementary material 1
