# Supplementary material for: Tn1 transposition in the course of natural transformation enables horizontal antibiotic resistance spread in Acinetobacter baylyi
Source: Microbiology (Reading). 2020 Dec 3;167(1):001003. doi: 10.1099/mic.0.001003 (PMC8116780; doi:10.1099/mic.0.001003)
Supplement: Supplementary material 1 [file mic-167-003-s001.pdf]

Supplemental Information

Tn*1* transposition in the course of natural transformation enables horizontal antibiotic resistance spread in *Acinetobacter baylyi*

Julia Kloos[1] (ORCID: 0000-0002-8773-3463), Pål J. Johnsen[1] (ORCID: 0000-0002-6455-6433), Klaus Harms[1] (ORCID: 0000-0003-1454-0692)

[1] Microbial Pharmacology and Population Biology Research Group, Department of Pharmacy, Faculty of Health Sciences, UiT The Arctic University of Norway, Tromsø, Norway

Supplemental Information: Supplemental Results

Natural transformation by linear donor DNA substrates

Initial natural transformation experiments of *A. baylyi* wildtype by linear pJK2-PCR (see Results and Discussion) resulted in transformation ($[2.5\pm2]\times10^{-8}$, n=3) and transposition frequencies ($[8.0\pm6.1]\times10^{-9}$) indistinguishable from those observed with circular pJK2 DNA. Seven transposants were recovered and included in the Sanger sequencing analysis for chromosomal Tn*1* insertion locus and TSD confirmation (Fig. 2 and Fig. 4). As a control experiment with different linear donor DNA, we employed a *Bst*Z17I/*Nhe*I restriction digest of pJK2 (pJK2-digest). Both enzymes cleave the vector backbone of pJK2 but not the Tn*1*. With this donor DNA substrate, the transformation ($[2.1\pm1.0]\times10^{-9}$, n=3) and transposition frequencies (2.1×10^{-10}) dropped about 30 times compared with circular pJK2. One transposant was found and included in the analyses for Fig. 2 and Fig. 4.

We analyzed the respective linear DNA substrates by PCR and found that DNA spanning the inverse PCR product endpoints or covering the cleavage sites were still detectable, presumably due to template DNA remnants or double cleavage escapees. Such molecules can facilitate circularization in the cytoplasm when taken up together with a linear DNA molecule with two overlapping ends. Apparently, traces of circular DNA were sufficient to facilitate efficient transformation and transposition. To rule out this possibility, we instead purified the pJK2-PCR substrate by agarose gel electrophoresis. After purification, no template DNA was detectable by PCR. Subsequent transformation experiments were conducted with gel-purified pJK2-PCR donor DNA substrate (Results and Discussion).

Detection and verification of Tn*1* transposition in Δ*recBCD* Δ*sbcCD*

The stable establishment of pJK2 in *A. baylyi* Δ*recBCD* Δ*sbcCD* mutants impeded quantification of Tn*1* transposants in that strain. To investigate whether transposition occurred at all in the Δ*recBCD* Δ*sbcCD* mutant, we isolated genomic DNA from mixed ampicillin-resistant colonies. Chromosomally inserted transposons would transform cells with much greater efficiency through homologous recombination than through transposition (Fig. 3). The genomic donor DNA (1,000 ng ml^{-1}) transformed the *A. baylyi* wildtype

strain at a frequency of 1.1×10^{-7} (n=1). Forty isolates were stably ampicillin-resistant after repeated re-streaking on selective medium, and PCR analyses showed the presence of Tn*1* but absence of pJK2 vector backbone DNA. Sanger sequencing of purified genomic DNA from these isolates confirmed chromosomal insertion of Tn*1* and canonical TSD in all cases. We identified 14 unique chromosomal Tn*1* insertion sites with canonical TSD, and 10 of the 14 insertions were located around the proposed terminus of replication (Supplementary Table S3; Fig. 4).

Supplemental information: Tables and Figure

Supplemental Table S1. List of primers used in this study.

Name	Sequence 5'-3'	Comment	Reference/source
Tn1-f+r	AGGGGTCTGACGCTCAGTGG	construction pJK2; transformant characterization (pJK2)	this study
cat-r	CGCGGATCCTTACGCCCCGCCCTGCCACTCATCG	construction pJK2; transformant characterization (pJK2; pTn*4401*); linear donor DNA analysis (pJK2)	this study
p15A-r1	TTACAACTTATATCGTATGG	construction pJK2; transformant characterization (pJK2; pTn*4401*); linear donor DNA analysis (pJK2)	this study
cat-f	CTCCGCTAGCGCTGATGTCC	inverse pJK2-PCR	[1]
p15A-ori-f-c	GCGCTAGCGGAGTGTATACTGG	inverse pJK2-PCR	this study
pJK2-tnpR-del-f	GGACTGCTGGCTGGTTGAGAC	construction pJK7	this study
pJK2-tnpR-del-r	CGAAAGGGCCTCGTGATACGCT	construction pJK7	this study
xerC-down-f	CTTCTAGATCGTGCCCATCCAAGAGCAC	construction strain ADP1 Δ*xerC*	this study
xerC-down-r	AACTTGAGCTCAACCGTTTGTAGACC	construction strain ADP1 Δ*xerC*	this study
xerC-up-f	GTAACACTTGCCTGTGGTACAGG	construction strain ADP1 Δ*xerC*	this study
xerC-up-r	ATCTAGATGACTGTTCAGCGCTCCAGC	construction strain ADP1 Δ*xerC*	this study
xerC-ctrl	AGCTTGGTACACAGCTTTGG	confirmation strain ADP1 Δ*xerC*	this study
sbcD-up-f	GTAACAAGGCAGACAAGATCGGTTGC	donor DNA substrate pKH80-PCR	this study
sbcC-down-r	AACTTGAGCTCAATCTCGCCATAAACCGTTG	donor DNA substrate pKH80-PCR	this study
bla-ins-f	CTACACGACGGGGAGTCAGG	transposant characterization (pJK2)	this study
Tn1-tnpA-ins-f	AATGATGAAGATATCGCACG	transposant characterization (pJK2)	this study
KPC-A	CTGTCTTGTCTCTCATGGCC	transformant characterization (pTn*4401*)	[2]
KPC-B	CCTCGCTGTGCTTGTCATCC	transformant characterization (pTn*4401*)	[2]

Supplemental Table S2. Tn*1*-insertions into wildtype ADP1.

**Transposition event**[a, b]	**Donor DNA**	**TSD sequence**[c]	**Tn*1* insertion loci**	**Orientation *tnpA* gene**	**MIC AMP (µg/ml)**[d]	**Gene ID/CDS**[e]	***A. baylyi* ADP1 genetic loci**[e]	**Product**[e]
1	pJK2	TTGAA	317509-13	transcriptional	> 256	*htpG*	316092..318014	chaperone Hsp90, heat shock protein C 62.5
2	pJK2-PCR	TTTAA	1734433-37	transcriptional	nd	*hcaB*	1733920..1735371	hydroxybenzaldehyde dehydrogenase
3	pJK2	GAAAA	1747112-16	opposite transcriptional	nd	*aroQ*←<br>→ACIAD1740	1746721 ←<br>→ 1747804	3-dehydroquinate dehydratase, type II ←<br>→ conserved hypothetical protein
4	pJK2	TAAAG	1758500-4	transcriptional	nd	*cioA*	1757096..1758523	ubiquinol oxidase subunit I
5	pJK2 dig	TTATT	1774647-51	transcriptional	nd	ACIAD1766	1773033..1774679	ABC superfamily, membrane component; putative branched-chain amino acid permease protein
6	pJK2	AATTA	1777741-45	opposite transcriptional	> 256	ACIAD1770	1777596..1777790	hypothetical protein; putative membrane protein
7	pJK2-PCR	TTTAA	1778746-50	opposite transcriptional	nd	ACIAD1772	1778317..1778766	conserved hypothetical protein; putative signal peptide
8	pJK2	TAGGA	1779507-11	transcriptional	nd	ACIAD1773	1779134..1780402	putative transport protein (permease)
9	pJK2-PCR	TTAAT	1782766-70	transcriptional	nd	*otsB* ←<br>→ ACIAD1776	1782760 ←<br>→ 1783137	trehalose-6-phosphate phosphatase, biosynthetic ←<br>→ putative general stress protein 26
10	pJK2	TAATA	1801767-71	transcriptional	nd	ACIAD1794	1800814..1802439	hypothetical protein; putative membrane protein
11	pJK2	TAATA	1804802-6	transcriptional	> 256	ACIAD1796←<br>→ ACIAD1798	1804635 ←<br>→ 1804946	hypothetical protein; putative membrane protein ←<br>→ putative lipopolysaccharide modification acyltransferase
12	pJK2	ATAAG	1807351-55	opposite transcriptional	nd	ACIAD1800	1807210..1808064	putative transporter (formate/nitrite transporter family)
13	pJK2-PCR	TTTTA	1808134-38	transcriptional	nd	ACIAD1800 ←<br>→ ACIAD1801	1808064 ←<br>→ 1808407	putative transporter (formate/nitrite transporter family) ←<br>→ conserved hypothetical protein
14	pJK2	ATATT	1814852-56	opposite transcriptional	nd	ACIAD1807	1813741..1814901	putative dipeptidyl aminopeptidase/acylaminoacyl-peptidase
15	pJK2-PCR	GTATA	1815400-4	transcriptional	nd	ACIAD1808	1814916..1816058	putative monooxygenase
16	pJK2	TTGAA	1839405-9	transcriptional	nd	ACIAD1831	1839058..1839816	conserved hypothetical protein
17	pJK2	TCAAA	1848469-73	opposite transcriptional	nd	ACIAD1845	1848146..1848571	hypothetical protein
18	pJK2	TGTAA	1880874-78	opposite transcriptional	nd	ACIAD1888	1880716..1881318	putative oxidoreductase related to nitroreductase
19	pJK2	TACCA	1886221-25	opposite transcriptional	> 256	ACIAD1896	1886048..1886923	putative permease (drug/metabolite transporter)
20	pJK2	GATAA	1886284-88	opposite transcriptional	nd	ACIAD1896	1886048..1886923	putative permease (drug/metabolite transporter)
21	pJK2-PCR	TAATA	1888252-56	transcriptional	nd	ACIAD1897	1887046..1888446	putative transcriptional regulator (GntR family)
22	pJK2	TATAC	1890823-27	transcriptional	> 256	*moeA*	1890678..1891904	molybdopterin biosynthesis protein
23	pJK2	ATTTA	1892847-51	transcriptional	> 256	*moaE*	1892848..1893402	molybdopterin converting factor, large subunit
24	pJK2-PCR	TTTAA	1903133-37	transcriptional	nd	ACIAD1911	1902439..1903785	putative nitrate transporter transmembrane protein (MFS superfamily)
25	pJK2	TATAA	1908554-58	transcriptional	nd	*fdhD*	1908406..1909227	formate dehydrogenase formation protein
26	pJK2	ATATT	2749626-30	transcriptional	> 256	ACIAD2807	2749479..2749832	hypothetical protein
27	pJK2	ATCTA	2986131-35	opposite transcriptional	> 256	ACIAD3059	2986058..2986909	conserved hypothetical protein
28	pJK2	TTAAA	3091433-37		nd	ACIAD3161 ←<br>→ ACIAD3162	3090969 ←<br>→ 3091445	putative outer membrane porin, receptor for Fe(III)-coprogen, Fe(III)-ferrioxamine B and Fe(III)-rhodotrulic acid uptake (fhuE) ←<br>→ conserved hypothetical protein
29	pJK2	TTTTA	3166549-53	transcriptional	nd	*hemF* ←<br>→ ACIAD3253	3166370 ←<br>→ 3166583	coproporphyrinogen III oxidase ←<br>→ hypothetical protein, putative membrane protein

[a] transposition events 2 to 25 occurred in the proposed terminus region of the ADP1 chromosome
[b] transposition events 19 and 22 were identified from the same isolate
[c] TSD = target site duplication
[d] MIC for ampicillin in wildtype ADP1 = 2 µg ml^{-1}; nd = not determined
[e] GenBank CR543861.1

Supplemental Table S3. Tn*1*-insertions into ADP1 Δ*recBCD*.

Transposition event[a, b]	Donor DNA[c]	TSD sequence[d]	Tn*1* insertion loci	Orientation *tnpA* gene	Gene ID/CDS[e]	*A. baylyi* ADP1 genetic loci[e]	Product[e]
1	g(Δ*recBCD* +pJK2)	ATCTA	1415265-69	transcriptional	ACIAD1420	1415091..1415651	conserved hypothetical protein; putative carbonate dehydratase
2	g(Δ*recBCD* +pJK2)	TTCAT	1747969-73	transcriptional	ACIAD1740	1747804..1748688	conserved hypothetical protein
3	g(Δ*recBCD* +pJK2)	ATATC	1754805-9	transcriptional	ACIAD1746	1754767..1754967	hypothetical protein
4	g(Δ*recBCD* +pJK2)	TACAA	1805822-26	transcriptional	ACIAD1798	1804946..1806739	putative lipopolysaccharide modification acyltransferase
5	g(Δ*recBCD* +pJK2)	GATTC	1839072-76	transcriptional	ACIAD1831	1839058..1839816	conserved hypothetical protein
6	g(Δ*recBCD* +pJK2)	TGCAA	1850443-47	transcriptional	ACIAD1848[f]	1850257..1850544	hypothetical protein[f]
7	g(Δ*recBCD* +pJK2)	TTGTA	1852702-6	opposite transcriptional	ACIAD1852	1851833..1853137	putative phage-related protein
8	g(Δ*recBCD* +pJK2)	ATGTA	1852731-35	transcriptional	ACIAD1852	1851833..1853137	putative phage-related protein
9	g(Δ*recBCD* +pJK2)	TGTTA	1852865-69	transcriptional	ACIAD1852	1851833..1853137	putative phage-related protein
10	g(Δ*recBCD* +pJK2)	AATTA	1853262-66	transcriptional	ACIAD1853	1853134..1853412	putative phage-related protein
11	g(Δ*recBCD* +pJK2)	GAATA	1854930-34	transcriptional	ACIAD1854 ← → ACIAD1855	1854824 ← → 1854952	putative phage-related protein ← → hypothetical protein; putative membrane protein
12	g(Δ*recBCD* +pJK2)	ATTTA	2304216-12	opposite transcriptional	ACIAD2339	2303197..2304708	putative flavin-binding monooxygenase
13	g(Δ*recBCD* +pJK2)	TAATT	2508372-76	transcriptional	ACIAD2549	2507921..2508592	putative sarcosine oxidase gamma subunit protein (soxG)
14	g(Δ*recBCD* +pJK2)	TAAAC	3253108-13	transcriptional	ACIAD3348	3252295..3253185	conserved hypothetical protein

[a] transposition events 2 to 11 occurred in the proposed terminus region of the ADP1 chromosome
[b] transposition events 6 to 11 occurred in a prophagic region of ADP1 at 1.8 Mb, as described in Barbe *et al*., 2004 [3]
[c] genomic DNA isolated from ampicillin-resistant ADP1 Δ*recBCD* transformed by pJK2
[d] TSD = target site duplication
[e] GenBank CR543861.1
[f] identified as essential by de Berardinis *et al.* [4]

Supplemental Table S4. Sequences of Tn*1* target site duplications and adjacent chromosomal regions in the ADP1 chromosome.

**Transposition event**[a]	**chromosomal sequence**[b]
1	CTGCCAAAAATTCGCCACAGCTTGAATTATTTAAGAAAAAAGGCATTGAAGTATTACTGATGTCTGAACGTGTAGACGAATGGGCAATGAACTTC
2	TTCAAGTACACCCAACATACTGCCTTGCTGCGTTGGATTACCTGCTTTAAGTGAGGCTGTTTTTTGAATGAGCTTTTCAATAAAGCGATCTGCAA
3	ATGATTGAAATAGAAATGGGCTTACCCGTGAAATGAAATCAGTAAGAAAATCATCTTATTTGTAGGTTGTAAACCACTTTTCTCTACGAAAATAA
4	AATATGAAATGAAACCGTAAATGCAAACTGGATTCGTGCTAGTTCTAAAGCTGTCAGTCCTAAGGTCATGACTGCACCTCACATTTTATATTCGT
5	CTGTTGGTTCTTGTGATTTTGTTTATTCAAAAACGTCCACAAGGTTTATTTGCAATCAAAGGCCGTTTTGTGGAGTAAATAACAATGAAGATCGC
6	TTTTATTAATGGTAATTAGTTTCATTTTATTTTTCTGTGCTTTCTTAATTAAGCTAGAGTTTAAATTGAATATTATATTTTGGAAAAATTCTTAA
7	CGCAAGCGTTAAAGCAGATACAACACCAGTTGTCAAAATTAAGTTTTTAAACATATTTTTCTCCAATTTAATTTTATTGCTTTGTTCAAATTAAG
8	CGTTTGCTTTAATACTATTGCTCACTTTGCTCCCAGTTTTAGCATTAGGAACAACTGCACTGACCATGGCGGTGATTCTATTTTTCTTTGGCACT
9	GATCATTTTTTTTAAGGATATTATTGTTCATTTTATTCACATTAGTTAATACTTATAAATTAACATGGATGAAAAATGATCAAAAATAAAGCAGA
10	AGCCATGCATGGAGCAGTGCGAGGTTCTGGACAAGTTGTAGCAGATAATAAAGTTGCTGGTGCTTTGGAGCAAAGTAGACAATTAAACCAGTTAG
11	ACTTAGAAGGCTCTGCTGGTCAAATTTATCAATGAAAAAAAGTAATAATAATTGTGTGATTTAAAGCTGAAATGAGGCCTTTGATTGCCATGAGT
12	TTGGAAAAAATACTCGCTTAGAGAGGTTTGTCCTTGCCAAACCAGATAAGCAACTTCTACAGAGCCCACGACAACATGCGTAAAATCACCTAAAC
13	CGTTATTTGTTTTATCAAATAAAATAATGCAGAGTCAGCAAATAGTTTTATAGCTACTTTTAGATGCAAAATGTAATGTTTTTGATTTTAGATTG
14	GGGTTGAACACAGTTCAATTGATAATCCACTGAATGCTGGAGCGTATATTGCAGATTGGTTGGCAGAACATTTAAACGGAAAAACTTCAAAGTAA
15	CCAATGGCCATGAACAACAATTTGACTTAGTGATCGGAGCAGATGGTATACATTCAAAAATACGCCATCTTGTCTTTCCCGAACTTGCTGAAAGT
16	TGTTTTCATCATAATTTAAGTGCTTTGCTTTCTTTTCAAATTGCATTGAACTCTCATATGTAAAACATTTACAGCCTTTTGATTCTTTATGAGTA
17	AGCCAATGTACTAAAAAATACGGTACATCCAACTCAGCAAGGTTCTCAAACCCCAGTTTATAATATGCGGGAAACGATTGACCAAATCTGCATCA
18	TCCCATTCTGCATGTACTTTGTCATCTACAACTGGATTGTAATGCTGTAAAGATGCACCTACATTCTCTGCTGCTAATGCTGTCCATACAGCAAG
19	TGTAACTGCCCTACTGTAGCAATTCCCCCCTTTGATAAACCATAGTACCAAAAGAAAAATCCGATCAGCATACTAAATAGTGAAACGTATACGAG
20	ATCAGCATACTAAATAGTGAAACGTATACGAGTGCGATCAAACCTGATAATGAAAGTTTAGTGATTACGTCGTTAGAATAATTCCAAACCATAAT
21	GCCTAAAGCAGGTATTTTTCTATGGTGCGAATTGCCCACAGATATTAATATCCAAAAATTAATGTCATATTGCACTGAAAAGGGAGTAATTTTAG
22	GGACTGTTGCTTTGCAAGGCTTGAAACCTTGAGCTCCCCTTTTACTATACTTGCTTGCATTCGAATAAAACGTTCCCTTTTATCTTCTTTCATGC
23	TTCAGGTTTCATGCCTACATTTTTCATGGAGAGTCCCGCAAATAGATTTAATGCTGATGATTATGGTGATTGTGATGATGTTCTGAATGATGGTG
24	GATCATTTTTTTTAAGGATATTATTGTTCATTTTATTCACATTAGTTAATACTTATAAATTAACATGGATGAAAAATGATCAAAAATAAAGCAGA
25	GTTGTGTATGACTGTGTAGTAGAGGAATTCCCTGTTGCTTTAATCTATAACGGGATCTCTCATGCAGTCATGATGACAACGCCGCTCGATCTTGA
26	TATCTGCACATTCACTCATATCATCCAGAATTCCCAGCACATTAGATATTGTGTCTACCATGATCTGTTGAATGATCGCAAAAAATACATCATGT
27	TAAGACCTGAATAGACCATTTTGCCTCAAGGGCTTTTTTTAGTCTATCTACAATATAAACTGAACGATGCTGACCGCCTGTGCAGCCTATTGAAA
28	ACATATCATTTTTATGATCTGAAAATACTGTGACATTTTGACTTATTAAAACCAAATTCTGGAAAAAGCCTTTATGCCAACGATCCTGTCGTCGA
29	AGTGTAAACTGGCAATTTATGTCATGCTTATTTCACAAAGGTTTGTTTTACTCAATAAAATTTTTTGAATAGGCTTTCACATGTTTCACATCTTA
30	ATAACTCATAGCTGGTACATAAAAGTTTGCAGGTAAGTCGCGGCCATCTACCACAGCATTGTGGCGAATTGCGCTATTTGGGCCAATTTTGCAAT
31	TTCTACAAACATAAGATTTTCACCATTTAACTTGGCAAAAGCTTGTTCATCTGCACGTTTAACCGCAGTCTGTGTTGGTGTCTTTAGTGTATTTT
32	AGCATTTATGTTATTTCTAGCAGTACAATGTACTGCACAAGTTCAATATCACTTAGATTCAATGTCTGGCGAAGATATGCCAGTGCCAGATCAAA
33	TATTCTATTTATTTGGTGCATTTTCCTGTGATTGTTTTGTACTTATACAAGCCTTTCTCAGGGACCATCATGCATCCAGCAAATATTCAGGAGCA
34	CAGCTATAAAATAAAAGTTTCATTCATGACTTCATTCTCCAAATGGATTCATGGAAATCATCACTGGAAGACCTAACTGTGACAAGAATCTCGTT
35	ATTGCCCCCAATGATTAGCTGGACTGCCCGATCAAATATGATCTTTGCAACAAACTCGAATGCTTGTTCTTTATCATTAATCATTATTTGACACC
36	GATGTTGCCAAACATATTTTTCGAACGGAAACATCTTTTTAGTCTTTGTAATATTCGTTTCTGCTTCATGCCAGATGTAAACGCGAGCTGTTTTA
37	AACATCTTTTTAGTCTTTGTAATATTCGTTTCTGCTTCATGCCAGATGTAAACGCGAGCTGTTTTATCACCAGTTTTCTCCAGGTGAATGTGCTC
38	ACGTTGAGTAATACACCAAATCTCAATACCCCGCTTTCGCATTGTTGTTAGGTCTAGAATCTGTTTATCACGTTTAGAATTATGACGGCTAAACA
39	GAAATCCTGAAAGTGCAAGCAGTGCGATTGCGTCAGATGCCATAGAATTAGCTTGCTGTTGAAGTCTTGATATATAGACGTTGAGTGCAGTCAAA
40	GAGTAGGTTCATTTAATTAACCTCATATAGAAAACCTACCCATACGAATATGAGTAGGTAATTGGAATCAGCGACCGATGAATCCGAGAATCTTG
41	GCCATTGTGACCAAAGACGGTAAGCAAAGACATATCGATTGCCTGATTTATGGAACTGGATTTATTACAGATCCGCGTATCTATTTGAAGCATTT
42	CCAGCATCCATCTTCTTGCTGTATGGCTTGATTGGGTTGCTGCGGTAATTGAAATCCATAACTGCTCAAGTAGGCAGCAGCATCTTGCCCTCTAA
43	AGAATTTGCAAAATAATGACGTTTAATCTTTTCGGGATGAAACTGTAAACGCCTCACGGTGATCCCCAGTGCTTTGGGGCGGTTCTGATAAGCAG

[a] Tn*1*-insertions into wildtype ADP1 (1-29) and ADP1 Δ*recBCD* (30-43)

[b] target site duplication (five nucleotides; green) and sequences upstream and downstream of the duplication (45 nucleotides, black) in 5’-3’ direction of the top strand

Supplemental Figure S1. Consensus DNA sequence logo for Tn*1*-insertions into the ADP1 chromosome based on Supplemental Table S4; visualization using WebLogo [5].

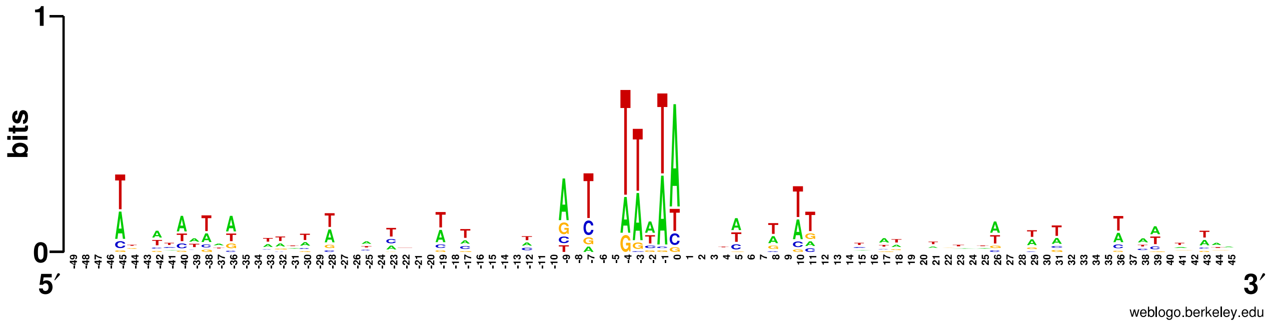

References

1. Domingues, S., et al., *Natural transformation facilitates transfer of transposons, integrons and gene cassettes between bacterial species.* PLoS Pathog, 2012. 8(8) DOI: e1002837.
2. Naas, T., et al., *Genetic structures at the origin of acquisition of the beta-lactamase bla KPC gene.* Antimicrob Agents Chemother, 2008. 52(4): p. 1257-63 DOI: 10.1128/AAC.01451-07.
3. Barbe, V., et al., *Unique features revealed by the genome sequence of Acinetobacter sp. ADP1, a versatile and naturally transformation competent bacterium.* Nucleic Acids Res, 2004. 32(19): p. 5766-79 DOI: 10.1093/nar/gkh910.
4. de Berardinis, V., et al., *A complete collection of single-gene deletion mutants of Acinetobacter baylyi ADP1.* Mol Syst Biol, 2008. 4: p. 174 DOI: 10.1038/msb.2008.10.
5. Crooks, G.E., et al., *WebLogo: a sequence logo generator.* Genome Res, 2004. 14(6): p. 1188-90 DOI: 10.1101/gr.849004.
